# Lactylation: The emerging frontier in post-translational modification

**DOI:** 10.3389/fgene.2024.1423213

**Published:** 2024-06-27

**Authors:** Zhou Lu, Xueting Zheng, Mingsong Shi, Yuan Yin, Yuanyuan Liang, Zhiyan Zou, Chenghe Ding, Yuanjing He, Yan Zhou, Xiaoan Li

**Affiliations:** ^1^ NHC Key Laboratory of Nuclear Technology Medical Transformation, Mianyang Central Hospital, School of Medicine, University of Electronic Science and Technology of China, Mianyang, China; ^2^ Department of Gastroenterology, National Clinical Key Specialty, Mianyang Central Hospital, School of Medicine, University of Electronic Science and Technology of China, Mianyang, China

**Keywords:** lactylation, lactate, post-translational modification, epigenetics, gene transcription

## Abstract

Lactate, a metabolic byproduct, has gained recognition as a highly influential signaling molecule. Lactylation, an emerging form of post-translational modification derived from lactate, plays a crucial role in numerous cellular processes such as inflammation, embryonic development, tumor proliferation, and metabolism. However, the precise molecular mechanisms through which lactylation governs these biological functions in both physiological and pathological contexts remain elusive. Hence, it is imperative to provide a comprehensive overview of lactylation in order to elucidate its significance in biological processes and establish a foundation for forthcoming investigations. This review aims to succinctly outline the process of lactylation modification and the characterization of protein lactylation across diverse organisms. Additionally, A summary of the regulatory mechanisms of lactylation in cellular processes and specific diseases is presented. Finally, this review concludes by delineating existing research gaps in lactylation and proposing primary directions for future investigations.

## 1 Introduction

Cellular metabolism produces numerous small molecules that serve as crucial substrates and sources of energy. Moreover, these small molecules can play a role in cell signaling and the regulation of gene expression. An important aspect of this process is the post-translational modification of proteins, which involves the covalent attachment of various chemical groups to amino acids. Histones, for example, can undergo multiple post-translational modifications (PTMs) such as methylation, acetylation, phosphorylation, and ubiquitination. In recent years, various novel histone modifications have been identified, including lactylation, isonicotinylation, crotonylation, benzoylation, and sulfation (Jiang Y. et al., 2021; Gao J. et al., 2023; Yu et al., 2023). Consequently, investigating the interaction between metabolites and proteins is crucial for comprehending diverse physiological and pathological processes, as well as for offering precise diagnostics and treatment options.

Lactate, a byproduct of glycolytic metabolism, has long been associated with low oxygen levels and considered a harmful metabolic waste product under hypoxic conditions (Notarangelo and Haigis, 2019). However, its important regulatory role in biological functions has not been widely recognized. Otto Warburg’s observation in the 1920s that cancer cells selectively metabolize glucose to lactate even under aerobic conditions, known as the Warburg effect, highlights the significance of lactate in cellular metabolism (Liberti and Locasale, 2020). Since then, there has been a significant focus on the biological function of lactate. Subsequent studies have demonstrated that lactate serves as a crucial energy source and signaling molecule, thereby playing a pivotal role in various physiological and pathological processes such as the regulation of inflammatory responses, wound healing, energy metabolism, and tumor development (Izzo and Wellen, 2019; Li et al., 2022).

Recent research by Zhao’s group, published in 2019, identified a new role of lactate in promoting histone modification. Like other PTMs, lactate can directly modify histones by adding lactyl group to lysine residues, which regulates gene expression and is involved in M1 macrophage homeostasis (Zhang et al., 2019). Further investigation has substantiated the significance of protein lactylation in the functionality of lactate, encompassing various biological processes. Nonetheless, the biological characteristics and regulatory elements of lactylation remain elusive. Here, we provide a thorough overview of lactate-induced lactylation, spanning from its genesis to its implications in diverse cellular processes and specific disease states.

## 2 Identification of lactylated proteins

The prevailing methods for analyzing lactylation involve mass spectrometry, immunoblotting, and computational prediction. Mass spectrometry utilizes liquid chromatography coupled with mass spectrometry to separate and detect peptides, enabling the identification of lactylation sites. Immunoblot analysis employs antibodies with specificity for lactylation motifs to detect lactylation modifications on specific proteins. Computational analysis utilizes machine learning models and bioinformatics tools to predict lactylation sites based on protein sequence and structural information. Lysine lactylation, identified as a prevalent PTM in nature, was initially detected through high-performance liquid chromatography (HPLC)-tandem mass spectrometry (MS/MS) analysis, revealing a mass increase of 72.021 Da on lysine residues and providing early evidence of histone lactylation. This discovery was further validated through the use of synthesized histone peptides with lysine lactylation modifications, pan anti-lactyllysine antibodies, and ^13^C-labeled lactate. The formation of cyclic ammonium ions of lactyllysine during MS/MS analysis serves as a new method for the identification of lysine lactylation (Wan et al., 2022). YnLac, a bioorthogonal chemical reporter functionalized with alkynyl groups, has also been developed for characterizing protein lactylation in mammalian cells (Sun Y. et al., 2022). An ultrasensitive lactate sensor may also be an important tool for identifying additional novel lactylated proteins (Li X. et al., 2023). In contrast to labor-intensive experimental techniques, the newly developed predictor FSL-Kla shows promise as a useful tool for the prediction of lactylation sites (Jiang P. et al., 2021). Subsequently, two novel computational models, Auto-Kla and DeepKla, were developed to predict protein lysine lactylation sites (Lv et al., 2022; Lai and Gao, 2023). These models have the potential to accelerate research in the field of protein modifications by providing a quick and reliable method to identify Kla sites, which could be instrumental in understanding the molecular mechanisms underlying various diseases and biological functions.

Numerous lactylated proteins and lactylation sites have been identified in various organisms, including mouse brain cells (Hagihara et al., 2021), *Trypanosoma brucei* (Zhang et al., 2021), *Botrytis cinerea* (Gao et al., 2020), rice grains (Meng et al., 2021), gastric cancer cells (Yang D. et al., 2022), *Frankliniella occidentalis* (An et al., 2022), HEK293 cells (Gaffney et al., 2020), *Phialophora verrucosa* (Song et al., 2022), and *Escherichia coli* (Dong H. et al., 2022). Notably, Lysine lactylation occurs in a diverse range of non-histone and histone proteins. Hence, the process of protein lactylation may play a role in the regulation of diverse biological processes. Discrepancies in the composition of lactylated proteins could stem from species-specific factors or the temporal and spatial dynamics of lactylation. Nevertheless, further investigation is needed to elucidate the specific biological functions of these lactylated proteins.

## 3 Molecular pathways of protein lactylation

**FIGURE 1:**
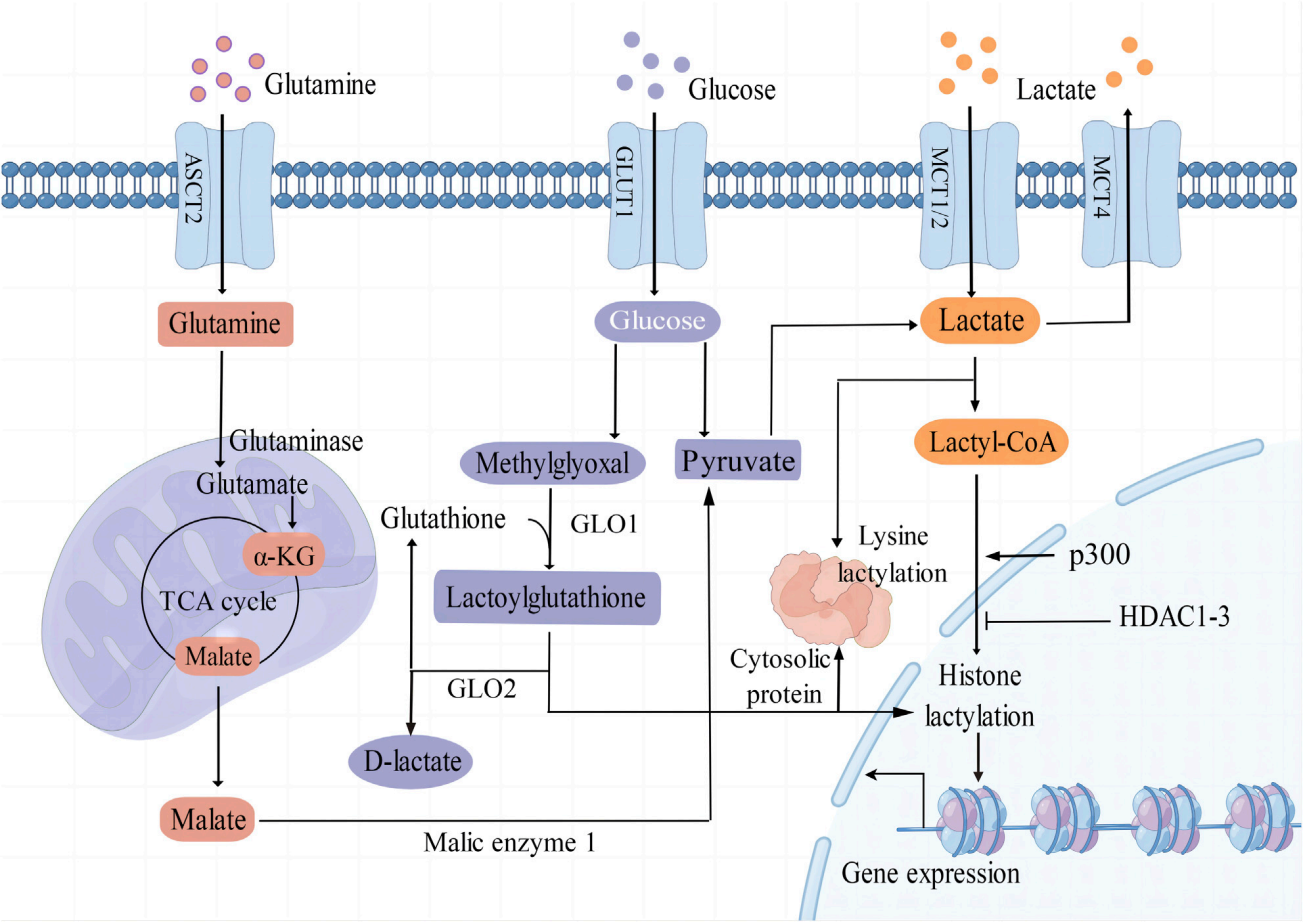
Different epigenetic mechanisms of protein lactylation. L-Lactate is an established precursor for lysine lactylation through lactyl-CoA. The main sources of lactate include glycolysis, glutamine catabolism, and lactate uptake mediated by MCT1/2. Additionally, methylglyoxal can be converted into lactoylglutathione and is involved in the lactylation of histones and non-histone proteins. GLUT1 glucose transporter 1; ASCT2 amino acid transporter type 2; MCT1/2/4 monocarboxylate transporter 1/2/4; α-KG α-ketoglutarate; GLO1/2 glyoxalase 1/2; p300 E1A binding protein p300; HDAC1-3 histone deacetylase 1–3; TCA tricarboxylic acid.

Lactate serves as a crucial substrate for the process of lactylation of both histone and non-histone proteins (Figure 1). It is predominantly generated as a byproduct of glucose metabolism through the glycolysis pathway. Monocarboxylate transporters (MCTs) play a key role in regulating the production and transport of lactate in and out of cells. MCT1 and MCT2 exhibit a high affinity for lactate, facilitating its uptake, while MCT4 is responsible for mediating the efflux of lactic acid (Brooks, 2018). Moreover, glutamine catabolism serves as an alternative pathway for lactate production in cancer cells. Specifically, glutamine undergoes a series of catalytic reactions to be converted into α-ketoglutarate (α-KG), which then enters the tricarboxylic acid (TCA) cycle. Within the TCA cycle, α-KG is further metabolized into malate and subsequently exits the mitochondria to be converted into pyruvate by malic enzyme in the cytoplasm (DeBerardinis et al., 2007).

Lactate can undergo conversion into lactyl-CoA and is implicated in the lysine lactylation of histones mediated by the acetyltransferase enzyme p300 (Zhang et al., 2019). Recent studies have shown that histone deacetylases 1–3 (HDAC1-3) are the main enzymes that remove histone lactyl modifications (Moreno-Yruela et al., 2022). Gaffney et al. (2020) proposed that lactylation involves a non-enzymatic acyl transfer process utilizing lactoylglutathione (LGSH) as a substrate. Overall, the precise mechanisms by which enzymes and accessory proteins regulate lactylation, including deposition, recognition, and removal of this modification, remain incompletely elucidated.

## 4 Function of protein lactylation

**FIGURE 2:**
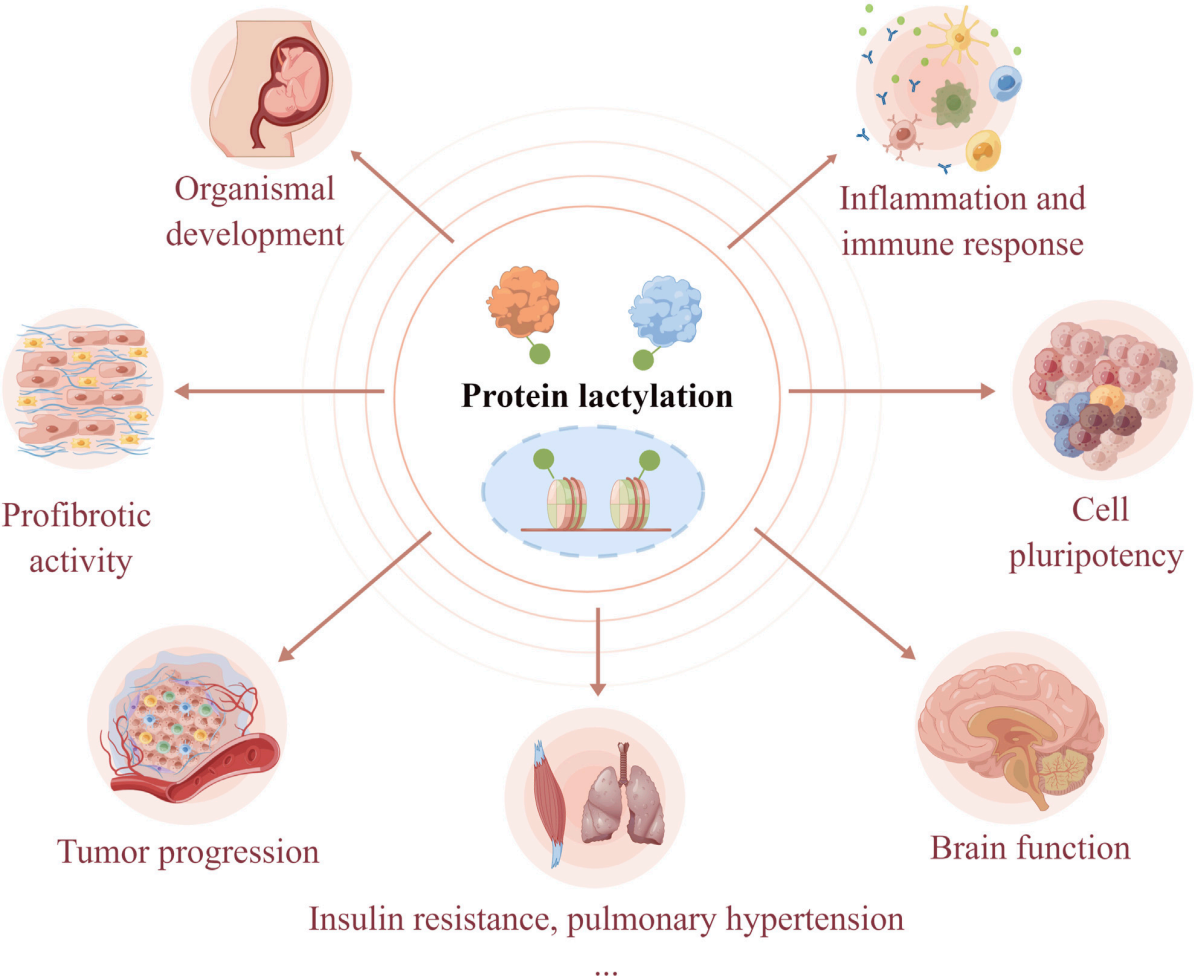
Lactylation is involved in the regulation of cellular physiological and pathological processes. As a novel post-translational modification, lactylation links cellular metabolism to epigenetic regulation. Lactylation has been shown to regulate organismal development, cell pluripotency, neural activities, tumorigenesis, immune response, profibrotic activity, insulin resistance, and hypoxic pulmonary hypertension.

Protein lactylation is an important way through which lactate performs its biological functions. Lactylation involves various cellular processes, including neural excitation, tumorigenesis, embryonic development, immunosuppression, pulmonary fibrosis, and metabolism (Figure 2). Moreover, the key substrates involved in lactylation and their corresponding physiological and pathological functions are summarized in Table 1. The function of protein lactylation discovered in recent years is summarized in the following sections.

**TABLE 1:** Key substrates of lactylation and their functions.

Protein	Sites	Function	References
Histone H3	K18	Regulates neural differentiation	Dai et al. (2022), Merkuri et al. (2024)
		Facilitates cellular reprogramming	Li et al. (2020)
		Promotes the stemness of glioblastoma cells	Li et al. (2023b)
		Potentiates Alzheimer’s disease	Wei et al. (2023)
		Accelerates tumorigenesis of ocular melanoma	Yu et al. (2021)
		Drives renal cell carcinoma progression	Yang et al. (2022c)
		Promotes colorectal tumorigenesis	Li et al. (2024a)
		Potentiates ocular melanoma progression	Gu et al. (2024)
		Suppresses bladder cancer progression	Xie et al. (2023)
		Promotes resistance to bevacizumab treatment	Li et al. (2023c)
		Promotes cisplatin resistance in bladder cancer	Li et al. (2024b)
		Promotes the progression of arsenite-related idiopathic pulmonary fibrosis	Wang et al. (2024)
		Promotes hepatic stellate cell activation	Rho et al. (2023)
		Promotes fibroblast-to-myofibroblast	Lin et al. (2024)
		Aggravates microvascular anomalies	Chen et al. (2024b)
Histone H3	K18, K23	Involves embryonic development	Yang et al. (2021), Yang et al. (2022b)
Histone H4	K5	Promotes PD-L1 expression	Huang et al. (2023)
HIF1α		Promotes angiogenesis and vasculogenic mimicry	Luo et al. (2022)
METTL16	K229	Promotes cuproptosis in gastric cancer	Sun et al. (2023)
DCBLD1	K172	Promotes cervical cancer progression	Meng et al. (2024)
p53	K120, K129	Contributes to tumorigenesis	Zong et al. (2024)
MRE11	K673	Enhances homologous recombination repair	Chen et al. (2024a)
AK2	K28	Facilitates tumor cell proliferation and metastasis	Yang et al. (2023)
PKM2	K62	Regulates macrophage phenotype transition	Wang et al. (2022b)
Ikzf1	K164	Promoters T_H_17 differentiation	Fan et al. (2023a)
METTL3	K281, K345	Strengthens immunosuppression of myeloid cells	Xiong et al. (2022)
MOESIN	K72	Enhances TGF-β signaling in Treg cells	Gu et al. (2022)
HMGB1		Increases endothelium permeability	Yang et al. (2022d)
Snail1		Promotes endothelial-to-mesenchymal transition	Fan et al. (2023b)
FASN	K673	Mediates hepatic lipid accumulation	Gao et al. (2023b)

### 4.1 Organismal development

Prior research has established the importance of epigenetic regulation in the development and growth of organisms (Frye et al., 2018; Furlong and Levine, 2018; Luo et al., 2018; Yadav et al., 2018). However, there is a paucity of studies examining the impact of lactylation on organismal development. A recent study indicated that histone H3 lysine 18 lactylation (H3K18la) correlates significantly with chromatin state and gene expression to favor neural differentiation (Dai et al., 2022). During neural crest cell development, histone lactylation plays a role in enhancing chromatin accessibility at active enhancers of critical genes (Merkuri et al., 2024). Moreover, lactate plays a role in promoting H3K18la in the endometrium, thereby regulating redox homeostasis and apoptotic balance to influence embryo implantation and uterine remodeling (Yang Q. et al., 2022). The evolving landscape of histone H3 lysine 23 lactylation (H3K23la), H3K18la, and pan histone lactylation has been characterized in oocytes and pre-implantation embryos. *In vitro* experiments have demonstrated that hypoxic conditions during culture hinder pre-implantation development by diminishing histone lactylation (Yang et al., 2021).

### 4.2 Cell pluripotency

Induced pluripotent stem cells play a critical role in the study of disease and regenerative medicine (Karagiannis et al., 2019). During the process of somatic cell reprogramming, Gli-like transcription factor 1 (Glis1) is directly involved in the modulation of chromatin structure, promoting closure at somatic genes while facilitating opening at glycolytic genes such as phosphoglycerate kinase 1 and hexokinase 2 (Li et al., 2020). In mouse embryonic stem cells, lactate has been shown to enhance the expression of genes associated with germline and cleavage embryos by inducing H3K18la (Tian and Zhou, 2022). Moreover, lactate produced by endothelial cells has been found to promote the differentiation of bone mesenchymal stem cells into osteoblasts and mitigate osteoporosis through the process of histone lactylation (Wu et al., 2023).

Cancer stem cells (CSCs) are believed to play a crucial role in tumorigenesis, recurrence, and therapy resistance, leading to higher recurrence rates and shorter overall survival. Hence, investigating the mechanisms by which CSCs maintain their stemness may provide a promising therapeutic approach for cancer patients. Hypoxia has been shown to induce β-catenin lactylation, which enhances its protein stability and promotes the stemness of colorectal cancer (CRC) cells (Miao et al., 2023). Additionally, the lactylation of H3 histone could regulate the self-renewal of glioblastoma cells through the MAP4K4/JNK/NF-κB pathway (Li L. et al., 2023). Similarly, H3 histone lactylation is elevated in liver cancer stem cells, efficiently facilitating the progression of hepatocellular carcinoma. However, the role of H3 histone lactylation in LCSCs requires additional research (Pan L. et al., 2022).

### 4.3 Brain function

Lactate plays a crucial role in various brain functions, such as providing energy, supporting neocortical development, regulating neuronal excitability, and maintaining homeostasis (Magistretti and Allaman, 2018; Dong X. et al., 2022; Medel et al., 2022). Increased lactylation is positively correlated with increased expression of the Fos proto-oncogene, heightened anxiety-like behavior, and reduced social behavior (Hagihara et al., 2021). Recent research has also observed elevated levels of histone lactylation in the brains of both Alzheimer’s disease mouse models and human patients. This lactylation is particularly elevated in microglia near Aβ plaque and is associated with increased expression of glycolytic genes, such as pyruvate kinase M2 (PKM2) and lactate dehydrogenase A (LDHA) (Pan R. Y. et al., 2022). Furthermore, it has been suggested that H3K18la may contribute to the progression of brain aging and Alzheimer’s disease pathology via the NF-κB signaling pathway (Wei et al., 2023). Intriguingly, physical exercise has been shown to induce a shift in microglial phenotype from pro-inflammatory to reparative through histone H3 lactylation, resulting in improved cognitive function and reduced neuroinflammation in mice (Han et al., 2023).

### 4.4 Tumor progression

Metabolic reprogramming and epigenetic remodeling are hallmarks of cancer and are tightly linked (Sun L. et al., 2022; Hanahan, 2022). Lactate from glycolysis contributes to tumor growth by promoting protein lactylation, which affects gene transcription and signaling pathways in various types of cancer cells. In ocular melanoma cells, elevated histone lactylation could promote the transcription of YTH N6-methyladenosine RNA binding protein F2 (YTHDF2) (Yu et al., 2021). Inactive von Hippel-Lindau (VHL) promotes the progression of clear cell renal cell carcinoma by initiating a positive feedback loop between histone lactylation and platelet-derived growth factor receptor β (PDGFRβ) signaling (Yang J. et al., 2022). Recent studies have demonstrated that lactylation modification plays a significant role in promoting tumor progression in various types of cancer, including prostate cancer (Luo et al., 2022), colorectal cancer (Wang et al., 2022a; Li X. M. et al., 2024), ocular melanoma (Gu et al., 2024), gastric cancer (Sun et al., 2023), bladder cancer (Xie et al., 2023) and cervical cancer (Meng et al., 2024). By binding to lactate, alanyl-tRNA synthetase 1 (AARS1) is able to catalyze the formation of lactate-AMP complex, thereby promoting p53 lactylation and tumor growth (Zong et al., 2024). Furthermore, the accumulation of lactate, induced by the transcription factor STAT5, promotes E3-binding protein nuclear translocation, leading to increased lactylation at the PD-L1 promoter and subsequent induction of PD-L1 transcription in leukemic cells (Huang et al., 2023). Additionally, histone lactylation has been implicated in drug resistance in colorectal cancer and bladder cancer (Li W. et al., 2023; Li F. et al., 2024). MRE11 lactylation has been shown to contribute to chemotherapy resistance by promoting homologous recombination repair (Chen Y. et al., 2024).

Currently, the impact of protein lactylation on the biological functions of tumor cells remains incompletely understood. Lactylated proteins and specific lactylation sites have been identified in many human tumors. Yang et al. identified 9,275 Kla sites and 9,140 proteins from tumor and adjacent liver samples. Notably, these sites are predominantly found on non-histone proteins, particularly enzymes associated with diverse metabolic pathways. Further investigations have indicated that lactylation of adenylate kinase 2 (AK2) lysine 28 diminishes enzymatic function, thereby promoting tumor cell proliferation and metastasis (Yang et al., 2023). Furthermore, 2,375 lysine lactylation sites in 1,014 proteins were identified in gastric cancer cells, with heightened lysine lactylation levels in gastric tumors correlating with a poorer prognosis (Yang D. et al., 2022). In non-small cell lung cancer (NSCLC), lactate can modulate glycolysis, mitochondrial homeostasis, and cell proliferation by affecting the expression of relevant genes through histone lactylation. However, the precise mechanism by which histone lactylation governs the expression of these genes remains unknown (Jiang J. et al., 2021).

### 4.5 Inflammation and immune response

Lactate is now recognized as a potent signaling molecule in inflammation and immune response. Increased lactylation is associated with macrophage phenotype in a time-dependent manner, thereby enhancing the expression of genes associated with tissue repair (Zhang et al., 2019; Irizarry-Caro et al., 2020; Wang et al., 2022b). Consistently, lactic acid-producing *Saccharomyces cerevisiae* has been shown to markedly reduce the expression of proinflammatory cytokines in dextran sulfate sodium (DSS)-induced mouse colitis (Sun et al., 2021). However, a separate study indicated that histone lactylation is not directly linked to alterations in macrophage activation state and gene expression during tissue repair. Instead, the induction of Arg1 by lipopolysaccharide (LPS) is reliant on interleukin-6 (Dichtl et al., 2021). Further research is necessary to explore potential associations between histone lactylation and Arg1 expression in macrophages. A recent study showed that lactylation is a crucial regulatory mechanism for CD4^+^ T-cell differentiation, specifically driving T helper 17 (T_H_17) differentiation in experimental autoimmune uveitis (Fan W. et al., 2023).

The glycolytic pathway is utilized by tumor cells to convert glucose to lactate, leading to immunosuppression and tumor progression. Lactate accumulation in the tumor microenvironment (TME) increases the expression of methyltransferase-like 3 (METTL3) through H3K18la in tumor-infiltrating myeloid cells, enhancing their immunosuppressive functions and promoting immune evasion. Mechanistically, METTL3 modifies Janus kinase 1 (Jak1) mRNA through N6-methyladenosine, leading to STAT3 activation and the expression of downstream genes (Xiong et al., 2022). Moreover, lactate could enhance regulatory T (Treg) cell function through lactylation of moesin at the lysine 72 residue, which enhances moesin interaction with transforming growth factor β receptor I (TGF-β RI) and downstream SMAD family member 3 (SMAD3) signaling (Gu et al., 2022).

Clinically, several clinical studies have explored the relationship between histone lactylation and inflammatory levels. During polymicrobial sepsis, lactate could promote high mobility group box-1 (HMGB1) lactylation in a p300/CBP-dependent mechanism and stimulate HMGB1 acetylation through G protein-coupled receptor 81 (GPR81) signaling. The subsequent release of lactylated/acetylated HMGB1 from macrophages through exosome secretion disrupts endothelium barrier function (Yang K. et al., 2022). Another clinical study reported that H3K18la may serve as a biomarker for the diagnosis and prognostication of septic shock (Chu et al., 2022).

### 4.6 Profibrotic activity

Augmented glycolysis is increasingly acknowledged as a significant factor in the development of fibrosis. Myofibroblast glycolysis induces p300-mediated histone lactylation and the expression of profibrotic genes in lung macrophages, thereby contributing to the pathogenesis of lung fibrosis (Cui et al., 2021). The crosstalk between alveolar epithelial cells and myofibroblasts through H3K18la facilitates the progression of arsenite-induced idiopathic pulmonary fibrosis (Wang et al., 2024). During hepatic stellate cell activation, hexokinase 2 (HK2) has been found to induce liver fibrosis by promoting histone lactylation (Rho et al., 2023). Following myocardial infarction, elevated lactate levels facilitate endothelial-mesenchymal transition via snail family transcriptional repressor 1 (Snail1) lactylation, leading to heightened cardiac fibrosis (Fan M. et al., 2023). Scleral glycolysis could promote myopia by driving fibroblast-to-myofibroblast transdifferentiation via H3K18la (Lin et al., 2024). Furthermore, pancreatic ductal adenocarcinoma is distinguished by a dense fibrotic stroma, with lactate secretion from neoplastic cells potentially inducing alpha-ketoglutarate (a-KG) production in mesenchymal stem cells. Subsequently, a-KG activates TET demethylase enzymes, promoting hydroxymethylation and reducing cytosine methylation in the process of *de novo* differentiation of MSCs into cancer-associated fibroblasts (Bhagat et al., 2019).

In addition to the function referred to above, lactylation is also associated with skeletal muscle metabolism, hypoxic pulmonary hypertension, nonalcoholic fatty liver disease, and diabetic retinopathy (Maschari et al., 2022; Gao R. et al., 2023; Chen et al., 2023; Chen X. et al., 2024). Overall, these studies have underscored the significant involvement of lactylation in various physiological and pathological pathways. Nevertheless, our comprehension of protein lactylation and its underlying biological processes remains limited.

## 5 Conclusions and perspectives

Lactylation of proteins is a recently discovered post-translational modification that exerts significant influence on various biological processes through intricate mechanisms. Lactate, serving as a crucial substrate for lysine lactylation, is implicated in numerous physiological and pathological processes such as traumatic brain injury, cardiovascular disease, respiratory disease, chronic liver disease, kidney disease, arthritis, and radioresistance (Yang et al., 2020; Li et al., 2022; Mao et al., 2024). The specific involvement of lactylation in these lactate-regulated processes remains to be elucidated. Moreover, lactic acid and its transporters have emerged as promising therapeutic targets for certain diseases. Presently, MCT1 inhibitor AZD3956 is being tested in a clinical trial (NCT01791595), and drugs targeting LDHA and MCT1 are under evaluation in preclinical research (Benjamin et al., 2016; Khan et al., 2020; Wang et al., 2021). Likewise, lactylation exhibits significant therapeutic promise as a target for inflammation, cancer, and other diseases. Further understanding of the diverse functions and molecular mechanisms of lactylation in regulating the aforementioned biological processes is imperative for expanding our comprehension of these processes and their potential clinical applications.

Despite the significant role that lactylation modification plays in various biological processes, research in this area is still in its early stages. There remain important unresolved issues and unexplored aspects that require further investigation. Increasing evidence indicates that lactylation may play distinct roles within varying cell types, potentially elucidating the diverse mechanisms and functions of lactylation across different cellular contexts. Nevertheless, the precise molecular mechanisms underlying protein lactylation and the downstream effector molecules are not yet fully elucidated. Additional studies are required to identify and characterize the enzyme responsible for the production of the intermediate lactyl-CoA and the regulatory proteins of lactylation, including specific writer, reader, and eraser proteins. Previously, many studies have focused on histone lactylation, but the function of this modification on non-histones is still unknown. Notably, CPLM 4.0, an updated database of protein lysine modifications (PLMs), provides a comprehensive resource on lactylation modifications. Its detailed annotations and integration of data from multiple resources provide a powerful foundation for further analysis of molecular mechanisms and regulatory roles of lactylation (Zhang et al., 2022). In conclusion, the discovery of protein lactylation not only opens a new field for the investigation of PTM but also brings new insight into the biological function of lactate. Lactylation has great potential as a therapeutic target and diagnostic biomarker in numerous diseases.
